# A Comparative Study of Consistency on 1.5-T to 3.0-T Magnetic Resonance Imaging Conversion

**DOI:** 10.2174/0115734056383931250919073319

**Published:** 2025-10-02

**Authors:** Jie Li, Yujie Zhang, Jingang Chen, Weiqi Liu, Yizhe Wang, Zhuozhao Zheng

**Affiliations:** 1 Department of Radiology, Beijing Tsinghua Changgung Hospital, School of Clinical Medicine, Tsinghua Medicine, Tsinghua University, Beijing, 102218, China; 2 Sophmind Technology (Beijing) Co., Ltd., Beijing 100083, China

**Keywords:** Magnetic resonance imaging, Brain segmentation, Repeated measures analysis of variance, Interclass correlation coefficient, Cicchetti's criteria, Signal-to-noise ratio

## Abstract

**Purposes:**

Deep learning methods were employed to perform harmonization analysis on whole-brain scans obtained from 1.5-T and 3.0-T scanners, aiming to increase comparability between different magnetic resonance imaging (MRI) scanners.

**Methods:**

Thirty patients evaluated in Beijing Tsinghua Changgung Hospital between August 2020 and March 2023 were included in this retrospective study. Three MRI scanners were used to scan patients, and automated brain image segmentation was performed to obtain volumes of different brain regions. Differences in regional volumes across scanners were analyzed using repeated-measures analysis of variance. For regions showing significant differences, super-resolution deep learning was applied to enhance consistency, with subsequent comparison of results. For regions still exhibiting differences, the Intraclass Correlation Coefficient (ICC) was calculated and the consistency was evaluated using Cicchetti's criteria.

**Results:**

Average whole-brain volumes for different scanners among patients were 1152.36mm^3^ (SD = 95.34), 1136.92mm^3^ (SD = 108.21), and 1184.00mm^3^ (SD = 102.78), respectively. Analysis revealed significant variations in all 12 brain regions (p<0.05), indicating a lack of comparability among imaging results obtained from different magnetic field strengths. After deep learning-based consistency optimization, most brain regions showed no significant differences, except for six regions where differences remained significant. Among these, three regions demonstrated ICC values of 0.868 (95%CI 0.771-0.931), 0.776 (95%CI 0.634-0.877), and 0.893 (95%CI 0.790-0.947), indicating high reproducibility and comparability.

**Discussion:**

This study demonstrates a deep learning-based harmonization method that effectively mitigates field strength-related inconsistencies between 1.5-T and 3.0-T MRI, significantly enhancing their comparability. The high ICCs observed in key brain regions confirm the robustness of this approach, paving the way for reliable clinical application across different scanners. A noted limitation is its current focus on brain imaging, which warrants future research to extend its applicability to other anatomical areas.

**Conclusion:**

This study employed a novel machine learning approach that significantly improved the comparability of imaging results from patients using different magnetic field strengths and various models of MRI scanners. Furthermore, it enhanced the consistency of central nervous system image segmentation.

## 1 INTRODUCTION

Magnetic resonance imaging (MRI) is widely used in neuroimaging due to its non-invasive nature, high soft-tissue contrast, and the availability of safe intracellular contrast agents [1]. Currently, common clinical MRI systems are classified into 1.5-Tesla (T) and 3.0-T scanners [2], primarily distinguished by their magnetic field strengths. The 1.5-T short-diameter MRI scanners are considered the standard for clinical applications [3]. However, 3.0-T scanners and increasingly, 7.0-T scanners are gaining popularity [4, 5]. This is because 3.0-T scanners offer a higher signal-to-noise ratio (SNR) and improved spatial resolution for distinguishing gray and white matter compared to 1.5-T scanners [6, 7]. As a result, images produced by 3.0-T scanners are generally sharper and provide greater detail [8].

Variations in scanning parameters or atlas templates used during image acquisition can lead to inconsistencies in whole-brain morphological findings across studies of the same disease [9]. While the availability of 3.0-T MRI has increased significantly over the past decade [10], most clinical scanners in clinical examination are still 1.5-T systems [11, 12]. As patients’ needs and MRI scanner availability increase, the demands for MRI applications continue to grow [13]. It was estimated that 60 million scans would be performed worldwide each year [14].

In addition, many previous longitudinal studies [15-17], such as the Alzheimer's Disease Neuroimaging Initiative (ADNI) project, utilized 1.5-T scanners during their initial phases [18]. To ensure the comparability of scan results, it is generally more effective to use the same scanner with identical parameters for the same patient during follow-up visits. However, this practice has limited the broader application of medical imaging and affected diagnostic accuracy. Improving the quality of 1.5-T MRI images to match the clarity and detail of 3.0-T images, while ensuring consistency across different scanners, would greatly enhance clinicians’ ability to make informed diagnostic and treatment decisions, thereby advancing clinical applications

To address the challenges of generalization and consistency in recognizing images from 1.5-T MRI scanners, this study utilized a super-resolution deep learning approach based on Convolutional Neural Networks (CNNs) to conduct harmonization analysis of whole-brain scans acquired from both 1.5-T and 3.0-T scanners [19]. Furthermore, the obtained analysis results were subjected to differential analysis and reproducibility analysis. A comparison was made between the differential analysis results obtained from the harmonized images and those from the non-harmonized images to evaluate the ability of the deep learning model to enhance the comparability of whole-brain 1.5-T MRI images to 3.0-T MRI images.

## 2 MATERIALS AND METHODS

### 2.1 Study Population

The study was a single-center retrospective one. The study population was adult subjects, and the project had been approved by the Ethics Committee of Beijing Tsinghua Changgung Hospital (No.22307-4-01). All subjects undergoing MRI scans between August 2020 and March 2023 were evaluated for inclusion and exclusion in the study based on the following criteria. The inclusion criteria were as follows: (1) patients aged 20–60 years; (2) normal vital signs and physical examination results during the screening period; and (3) completion of scans on both 1.5-T and 3.0-T MRI scanners within a three-week period. Additionally, patients with intracranial diseases were excluded, including tumors, cerebrovascular diseases, infections, and postoperative treatments. The gender distribution of subjects in each group was controlled at a 1:1 ratio.

### 2.2 Experimental Equipment

In this study, all the patients underwent whole-brain MRI scans by the scanners of 3.0-T Philips, 3.0-T GE (General Electric Company Healthcare), and 1.5-T UIH (United-Imaging Healthcare Technology) in the Radiology Department of Beijing Tsinghua Changgung Hospital. The voxel size for three-Dimensional T1-Weighted (3D-T1) Sequence and three-Dimensional Fluid-Attenuated Inversion Recovery (3D-FLAIR) Sequence were both set to 1*1*1 mm^3^, Magnetic Resonance Angiography (MRA) was the standard sequence. The specific MRI scan parameter settings for 3D-T1, 3D-FLAIR, and MRA are as follows: (1)3D-T1:TR 6.6 ms, TE 3.0 ms, TI 450 ms, FOV 240 mm* 240mm;(2)3D-FLAIR: TR 4800ms, TE 299 ms, TI 1650 ms, flip angle 90°, FOV 240 mm* 240mm; (3)MRA: TR 20 ms, TE 2.5 ms, flip angle 20°, slice thickness 1.4 mm, FOV 240 mm* 240mm.

### 2.3 Outcome Measures

An automatic algorithm (FSL-FAST) was used to segment the brain images obtained by different scanners [20]. The whole brain volume was used as global quantitative indicators. We used the volume of each subnucleus of cerebral parenchyma, gray matter, white matter, cerebral cortex, frontal lobe, temporal lobe, parietal lobe, occipital lobe, paracentral lobule, basal ganglia, limbic system, cingulate gyrus, cerebellum, anterior cingulate gyrus, corpus callosum, and ventricle were segmented by automatic algorithm as regional quantitative indicators.

### 2.4 Statistical Analysis

In this study, “repeated measurement analysis of variance” (ANOVA) [21] was used to evaluate whether there were differences in the segmentation results of brain images obtained by different MRI scanners. For the brain regions with differences, A super-resolution deep learning method was used to optimize the consistency of the MRI scan results [22], and then the differences between the results were compared. For the brain regions that still had differences, the Intraclass Correlation Coefficient (ICC) of measurement results of different instruments and equipment was calculated by using R software (version 4.2.2), IRR package, and the consistency degree was evaluated by Cicchetti four-levels standard [19]. The evaluation criteria were: (1) Level 1 (Chance Agreement) < 0.500; (2) 0.500 ≤ Level 2 (Systematic but Not Significant Agreement) < 0.750; (3) 0.750 ≤ Level 3 (Significant Agreement) < 0.900; (4) Level 4 (Near Perfect Agreement) ≥0.900. Hypothesis tests were 2-sided, and p less than 0.05 were considered statistically significant.

## 3 RESULTS

### 3.1 Whole Brain Segmentation of MR Scan Images

A total of 30 participants were included in this study, and each patient was scanned using Philips, GE, and UIH MRI scanners, and finally obtained a total of 90 sets of images. Among them, the mean whole brain volume obtained by Philips was 1152.36mm^3^ [Standard Deviation (SD) = 95.34], and the mean whole brain volume obtained by GE was 1136.92mm^3^ (SD = 108.21), the UIH device is 1184.00mm^3^ (SD = 102.78), and whole brain segmentation was performed for each image to divide the volume of each brain region (Table **1**).

The differences among different MRI scanners were compared for the various brain regions delineated. The differences between the Philips scanner (3.0-T) and the GE scanner (3.0-T) were assessed using ANOVA. The results indicated that, with the exception of the cerebellum region [F (1, 29) = 6.159, p = 1.911e-02, η_p_^2^ = 0.175], corpus callosum region [F (1, 29) = 5.269, p = 2.913e-02, η_p_^2^ = 0.154], and ventricle region [F (1, 29) = 6.356, p = 1.745e-02, η_p_^2^ = 0.180], there were no significant differences in the measurements among the 3.0-T scanners for the remaining brain regions (Table **2**).

Subsequently, repeated measurement ANOVA was used to compare the differences in the division of brain regions among the three MRI scanners of Philips (3.0-T), GE (3.0-T), and UIH (1.5-T) (Table **2**). If the assumption of Mauchly's Test for Sphericity (statistic W, p < 0.05) was violated among the levels of the independent variable, the results were adjusted using the Greenhouse-Geisser epsilon (GGe) from the Sphericity Corrections. As shown in Table **2**, significant differences (p < 0.05) were observed in twelve brain regions, including the aforementioned three regions. These findings suggest substantial variations in imaging results obtained from different magnetic field strengths, indicating a lack of comparability across devices for the same individual.

### 3.2 Segmentation of Brain Regions after Consistent Optimization

**Fig. (1):**
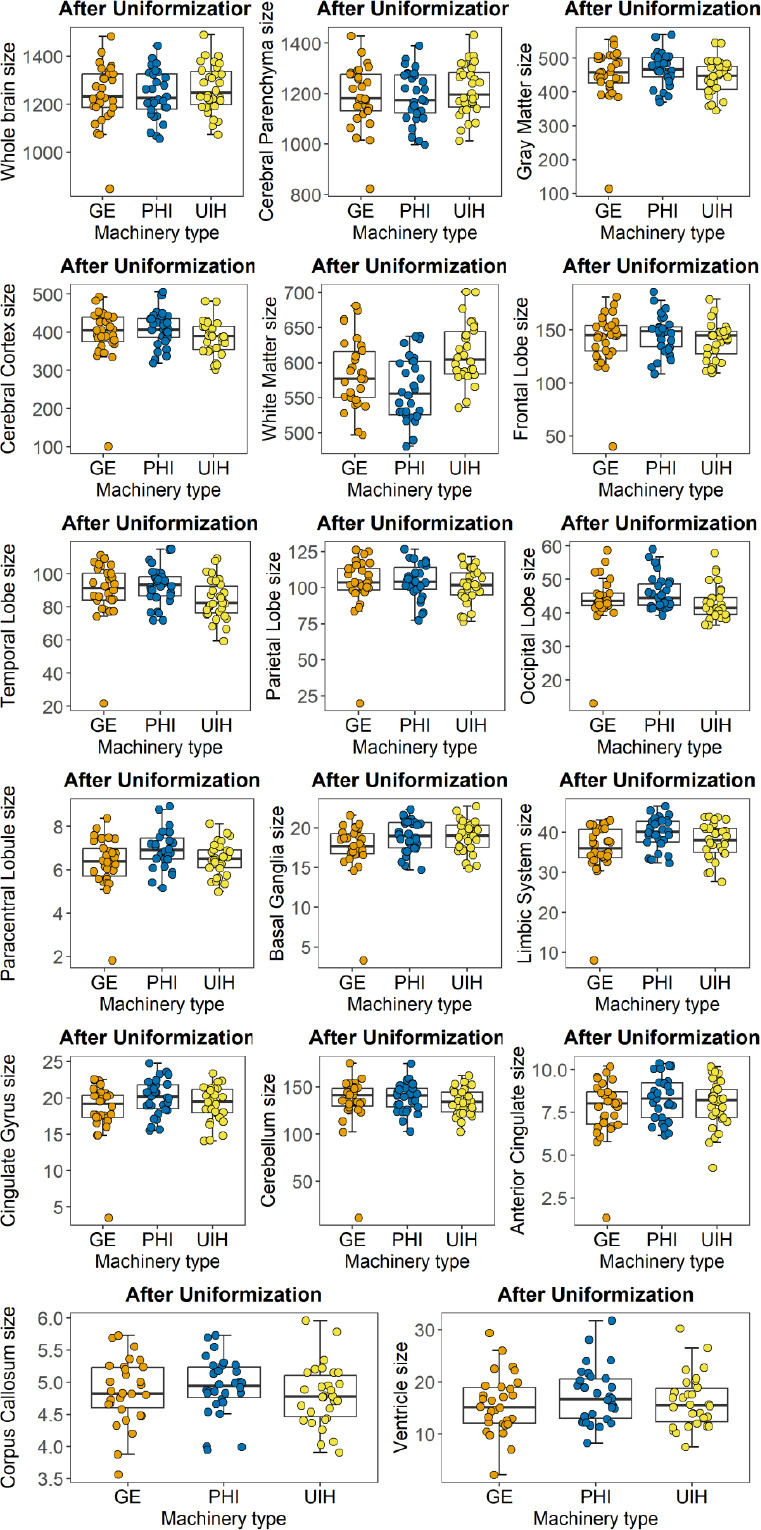
The segmentation results of brain regions in different machineries after uniformization. x-axis: Volumes of different brain regions (mm^2^); y-axis: Different MRI scanners.

A super-resolution deep learning algorithm was used to optimize the consistency of the MRI scan results (Fig. **1**). For the aforementioned 12 brain regions with existing differences, a differential analysis using repeated measures ANOVA was re-conducted (Table **3**). The results indicated that there were no significant differences (*p* > 0.05) among the three instruments for the segmentation results of the whole brain region [F (2, 58) = 3.728, p = 0.061, η_p_^2^ = 0.114], gray matter region [F (2, 58) = 2.885, p = 0.098, η_p_^2^ = 0.090], cerebral cortex region [F (2, 58) = 3.015, p = 0.091, η_p_^2^ = 0.094], frontal lobe region [F (2, 58) = 2.475, p = 0.123, η_p_^2^ = 0.079], parietal lobe region [F (2, 58) = 0.491, p = 0.500, η_p_^2^ = 0.017], and cerebellum region [F (2, 58) = 0.9078, p = 0.353, η_p_^2^ = 0.030]. These findings demonstrate that, following consistency analysis, scan results from MRI scanners with varying magnetic field strengths were rendered comparable.

### 3.3 Interclass Correlation Coefficient Analyses

**Fig. (2):**
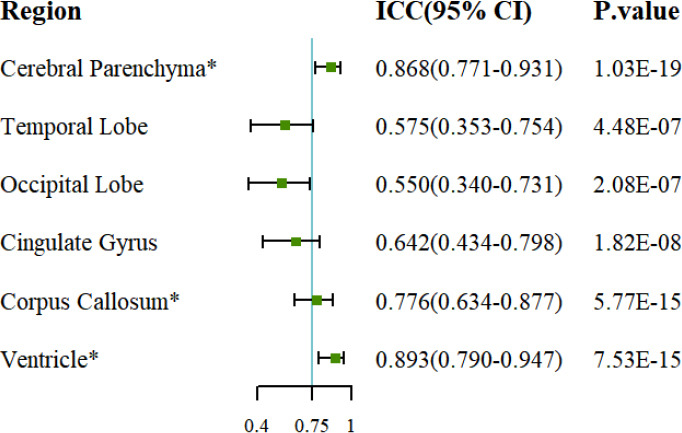
Intraclass correlation coefficient (ICC) among three MRI scanners. *ICC > 0.750, indicating well consistency. CI: Confidence Interval.

Although most of the brain regions showed no differences in the segmentation results after the consistency analysis, there were still significant differences observed in six brain regions. For these six regions, we conducted ICC analyses (Fig. **2**). The ICC values were 0.868 (95% CI 0.771-0.931) for the cerebral parenchyma region, 0.776 (95% CI 0.634-0.877) for the corpus callosum region, and 0.893 (95% CI 0.790-0.947) for the ventricle region. An ICC value above 0.750 indicates good reproducibility of the measurements. This suggested that even though there were differences in the results after harmonization across the three scanners, the results were reproducible, indicating comparability between the results.

## 4 DISCUSSION

This study recruited 30 eligible patients and obtained a total of 90 MRI scans after scanning with three MRI scanners. After segmenting the images into brain regions, among the 17 regions segmented by scanners with the same magnetic field strength (3-T), only three regions (cerebellum, corpus callosum, and ventricle) showed differences. However, for scanners with different magnetic field strengths, there were differences observed in 12 brain regions. Previous research findings have also indicated that there were statistically significant differences in imaging results between different field strengths in brain imaging, which aligned with the findings of this study [23-25].

After applying a machine learning algorithm for harmonization, the differential analysis showed no significant differences in the segmentation results of scanners with varying magnetic field strengths, except for the cerebral parenchyma, temporal lobe, occipital lobe, cingulate gyrus, corpus callosum, and ventricles. Similar to other related articles, utilizing various deep learning methods for image processing was found to improve the consistency and comparability of images acquired at different field strengths [26-28]. Subsequently, the ICC analyses were conducted for these six regions, and three of them exhibited high measurement reproducibility, indicating comparability among the results. Although previous research showed consistency between images obtained at 1.5-T and 3.0-T field strengths, the research in question utilized MRI for the measurement of volumetric liver fat fraction (VLFF) and applied the HepaFat-Scan technique, which involved processing the raw measurements [29].

Previous research showed that 3.0-T scanner images produce more pronounced susceptibility artifacts due to differences in the magnetic susceptibility of tissues and materials (implants) inside the body [30]. Implants in the patient's body could cause images to have misregistration, distorted, or blackened areas [31]. Susceptibility artifacts could still appear in images generated by the 1.5T scanner, but they were less noticeable, and the images obtained are still very diagnostic [32].

In addition, due to the enhanced magnetic field, the specific absorption rate (SAR) of the instrument was increased [33, 34], that is, the body absorbs more heat during the scan [6]. Therefore, the 3.0-T scanner was also more likely to reach the SAR limits specified by the human body and not be able to complete the scan [35]. When considering the limitations of the patient's artificial grafts, the heat absorption of the scanning process, and the cost of equipment maintenance, it was a better choice to rely on the 1.5-T scanner to obtain diagnostic images.

### 4.1 The Value of Clinical Application

This study enhanced the resolution and accuracy of 1.5-T MRI scanners, achieving consistency with the high-resolution imaging results of 3.0-T scanners. This approach not only addressed the primary limitations of 1.5-T scanners but also mitigated susceptibility artifacts commonly associated with 3.0-T scanners and reduced discomfort from excessive somatosensory heat during scanning. Consequently, this refined MRI analysis technique offers improved applicability for a broader population, including individuals with artificial implants.

Concurrently, the consistency and generalizability of the analysis results enhanced the comparability of measurements across different scanners and time points, enabling patients to undergo scans with instruments of varying parameters for each diagnosis, thereby improving diagnostic efficiency and ensuring the accuracy of diverse measurement outcomes.

### 4.2 Advantages and Limitations

This study applied a novel machine learning method to MRI scans of varying resolutions, enabling the analysis results from low-resolution scanners to align with those from high-resolution scanners, thereby offering significant clinical value, as previously discussed. In addition, this study compared image results from different brain regions, taking into account both global metrics to explore whole-brain indices and local segmentation for volume changes in specific brain regions, providing a comprehensive assessment of the brain. However, this study only explored the results of brain MRI imaging, and the imaging results of 1.5-T scanners in vascular imaging and spinal cord imaging were suboptimal. Whether this method could be extended to other tissues remains under investigation.

## CONCLUSION

In this study, the newly developed machine learning method was applied to improve the comparability between MRI imaging of patients with different magnetic field strengths and different types of MRI scanners, and the consistency with central nervous system image segmentation. In order to improve the diagnostic efficiency and universality of MRI imaging, it could provide a new direction for providing better quality services to the patients.

## Figures and Tables

**Table 1:** Brain segmentation of the patients.

	**PHILIPS ^a^** **(n=30)**	**GE ^a^** **(n=30)**	**UIH ^a^** **(n=30)**
Whole brain	1152.36±95.34	1136.92±108.21	1184.00±102.78
Cerebral Parenchyma	1100.15±96.38	1086.66±105.87	1132.79±103.71
Gray Matter	480.25±46.38	478.67±80.77	508.4±50.43
Cerebral Cortex	418.11±41.54	418.32±70.97	445.98±45.07
White Matter	469.16±42.44	464.29±55.02	471.8±46.22
Frontal Lobe	149.72±16.87	152.35±28.11	159.92±18.57
Temporal Lobe	98.16±10.30	98.12±14.80	104.37±12.53
Parietal Lobe	104.75±11.68	102.86±20.60	113.59±12.56
Occipital Lobe	44.37±4.65	43.92±6.57	46.43±4.55
Paracentral Lobule	6.95±0.85	7.11±1.55	7.26±0.83
Basal Ganglia	21.83±2.51	20.91±4.01	21.32±2.49
Limbic System	40.32±3.56	39.44±6.45	41.11±4.00
Cingulate Gyrus	19.65±2.22	19.39±3.85	20.52±2.37
Cerebellum	129.77±13.30	123.17±19.50	131.68±13.49
Anterior Cingulate	7.97±1.20	7.94±1.75	8.42±1.30
Corpus Callosum	4.21±0.43	4.1±0.46	4.1±0.42
Ventricle	18.93±5.63	17.71±6.36	17.94±5.45

**Note:** ^a^ Data are means ± standard deviations. The unit of measurement is mm^3^.

**Table 2:** Difference analysis of measurement results using MRI scanners.

	**Differences between GE (3T) and PHILIPS (3T)**				**Differences between 3 type of machineries (including 1.5T UIH)**							
	**F (1,29)**	**P_F_**	**ges^a^**	**η_p_^2 d^**	**F (2,58)**	**P_F_**	**ges^a^**	**W^b^**	**P_W_**	**GGe^c^**	**P_GGe_**	**η_p_^2 d^**
Whole brain	2.675	1.127e-01	0.006	0.845	17.452	1.166e-06	0.037	0.277	1.602e-08	0.581	1.034e-04*	0.376
Cerebral Parenchyma	2.256	1.200e-01	0.005	0.081	20.915	1.448e-07	0.036	0.345	3.427e-07	0.604	2.184e-05*	0.419
Gray Matter	0.017	8.968e-01	1.493e-04	0.001	5.582	6.068e-03	0.049	0.090	2.373e-15	0.524	0.023*	0.161
Cerebral Cortex	4.173e-04	9.838e-01	3.513e-06	1.439e-05	6.840	2.153e-03	0.057	0.097	6.203e-15	0.525	0.013*	0.191
White Matter	0.499	4.858e-01	0.003	0.017	0.862	4.278e-01	0.004	0.191	8.295e-11	0.553	0.371	0.029
Frontal Lobe	0.452	5.065e-01	0.003	0.015	5.341	7.429e-03	0.039	0.133	5.584e-13	0.536	0.026*	0.156
Temporal Lobe	3.871e-04	9.844e-01	2.630e-06	1.335	7.505	1.263e-03	0.053	0.402	2.839e-06	0.626	0.006*	0.206
Parietal Lobe	0.340	5.641e-01	0.003	0.012	9.345	3.035e-05	0.086	0.138	9.156e-13	0.537	0.004*	0.244
Occipital Lobe	0.251	6.203e-01	0.002	0.009	6.053	4.098e-03	0.042	0.238	1.833e-09	0.567	0.016*	0.173
Paracentral Lobule	0.568	4.51e-01	0.004	0.019	1.685	1.945e-01	0.013	0.201	1.700e-10	0.556	0.205	0.055
Basal Ganglia	1.882	1.807e-01	0.019	0.061	1.356	2.568e-01	0.015	0.150	3.019e-12	0.541	0.256	0.045
Limbic System	0.839	3.674e-01	0.007	0.028	2.035	1.399e-01	0.020	0.185	5.640e-11	0.551	0.163	0.066
Cingulate Gyrus	0.244	6.252e-01	0.002	0.008	3.440	3.876e-02	0.028	0.151	3.118e-12	0.541	0.070*	0.106
Cerebellum	6.159	1.911e-02*	0.039	0.175	8.052	8.201e-04	0.053	0.066	3.298e-17	0.517	0.008*	0.217
Anterior Cingulate	1.179e-02	9.143e-01	7.389e-05	4.065e-04	3.469	3.775e-02	0.024	0.260	6.609e-09	0.575	0.066	0.107
Corpus Callosum	5.269	2.913e-02*	0.016	0.154	4.037	2.283e-02	0.015	0.599	7.661e-04	0.714	0.037*	0.122
Ventricle	6.356	1.745e-02*	0.010	0.180	5.141	8.798e-03	0.008	0.176	2.793e-11	0.548	0.027*	0.151

^a^ges: η^2^,Generalized Eta-Squared. ^b^Mauchly's Test for Sphericity: When the P-value of the sphericity test is greater than 0.05, all levels of the independent variables conform to the sphericity hypothesis, that is, they are independent of each other. Otherwise, the Sphericity hypothesis is not met, and Sphericity Corrections are required. ^c^Greenhouse-Geisser epsilon: results of Sphericity Corrections. ^d^ Calculated by: SSn / (SSn + SSd). *means p<0.05.

**Table 3:** Repeated measures analysis of variance after normalization.

	**F (2,58)**	**P_F_**	**ges^a^**	**W^b^**	**P_W_**	**GGe^c^**	**P_GGe_**	**η_p_^2 d^**
Whole brain	3.728	3.000e-02	0.012	0.106	2.124e-14	0.528	0.061	0.114
Cerebral Parenchyma	4.114	2.134e-02	0.011	0.122	1.564e-13	0.532	0.049*	0.124
Gray Matter	2.885	6.393e-02	0.025	0.113	5.660e-14	0.530	0.098	0.090
Cerebral Cortex	3.015	5.681e-02	0.025	0.109	3.151e-14	0.529	0.091	0.094
Frontal Lobe	2.475	9.300e-02	0.016	0.166	1.255e-11	0.545	0.123	0.079
Temporal Lobe	7.924	9.073e-04	0.064	0.301	5.010e-08	0.589	0.006*	0.215
Parietal Lobe	0.491	6.145e-01	0.004	0.120	1.358e-13	0.532	0.500	0.017
Occipital Lobe	4.458	1.582e-02	0.042	0.167	1.320e-11	0.546	0.040*	0.133
Cingulate Gyrus	7.387	1.387e-03	0.051	0.230	1.144e-09	0.565	0.008*	0.203
Cerebellum	0.907	4.094e-01	0.012	0.094	4.077e-15	0.525	0.353	0.030
Corpus Callosum	3.183	4.880e-02*	0.015	0.933	0.376	0.937	0.052	0.099
Ventricle	8.891	4.28e-04	0.017	0.785	0.034	0.823	0.001*	0.235

**Notes:** ^a^ges: η^2^,Generalized Eta-Squared. ^b^Mauchly's Test for Sphericity: When the P-value of the sphericity test is greater than 0.05, all levels of the independent variables conform to the sphericity hypothesis, that is, they are independent of each other. Otherwise, the Sphericity hypothesis is not met, and Sphericity Corrections are required. ^c^Greenhouse-Geisser epsilon: results of Sphericity Corrections. ^d^ Calculated by: SSn / (SSn + SSd). * means p<0.05.

## Data Availability

The data and supportive information are available within the article.

## LIST OF ABBREVIATIONS

MRI: Magnetic Resonance Imaging
ICC: Interclass Correlation Coefficient
CNN: Convolutional Neural Networks
GE: General Electric Company Healthcare
UIH: United-Imaging Healthcare Technology
3D-T1: Three-Dimensional T1-Weighted
3D-FLAIR: Three-Dimensional Fluid-Attenuated Inversion Recovery
MRA: Magnetic Resonance Angiography
ANOVA: repeated measurement analysis of variance
SD: Standard Deviation

## STANDARDS OF REPORTING

STROBE guidelines were followed.
